# A Gene Panel for Early Identification of Future Responders to Immune Checkpoint Blockade

**DOI:** 10.3389/fgene.2022.706468

**Published:** 2022-03-03

**Authors:** Anshuman Panda, Anil Betigeri, Shridar Ganesan

**Affiliations:** ^1^ Rutgers Cancer Institute of New Jersey, New Brunswick, NJ, United States; ^2^ Akash Institute of Medical Sciences, Bangalore, India; ^3^ Rutgers Robert Wood Johnson Medical School, New Brunswick, NJ, United States

**Keywords:** immune checkpoint inhibitors, PD-1 blockade, CTLA-4 blockade, anti PD1/PDL1 immunotherapy, interferon gamma (IFN*γ*), cytotoxic T cell, PD1 blockade, CTLA4 blockade

## Abstract

Immune checkpoint blockade (ICB), therapies that target the PD-1 pathway, CTLA-4 pathway, and other checkpoint pathways, lead to durable responses in many cancer types. Since only a minority of patients respond to ICB, it may be useful to identify the future responders early in the course of treatment. In this study we evaluated a small (15 genes) biologically motivated panel, consisting of genes involved in immune activation and checkpoint pathways, for early identification of future responders to ICB. The panel passed consistency check, pathological and in-silico validations, and was an excellent predictor (area under ROC curve >0.95) of eventual response to ICB, both CTLA-4 and PD-1 blockade, when applied to metastatic melanoma patients undergoing ICB (i.e., “on-treatment”) in a publicly available dataset. These results suggest that this small biologically motivated panel may be useful for early identification of future responders to ICB.

## Introduction

Immune checkpoint blockade (ICB) leads to durable responses in many cancer types (Kyi and Postow, 2016; Hargadon et al., 2018; Arora et al., 2019). Since only a minority of patients respond to ICB and a majority of patients do not (Yarchoan et al., 2017), it may be useful to identify the future responders early in the course of treatment. In this study, we evaluated a small (15 genes) biologically motivated panel for early identification of future responders to ICB.

Based on the current understanding of the mechanism of action of immune checkpoint blockade (Tumeh et al., 2014; Anagnostou and Brahmer, 2015; Ribas, 2015), potentially sensitive tumors are expected to show evidence of immune activation and checkpoint pathway upregulation. Immune activation involves interferon gamma (*IFNG*) inducing movement of T cells (markers: *CXCL9*, *CXCL10*) towards target cells (Taub et al., 1993; Liao et al., 1995; Farber, 1997), which leads to infiltration of cytotoxic T cells (marker: *CD8A*) among target cells. Cytotoxic T cells make pores in plasma membranes of target cells using perforin (*PRF1*) and deploy granzyme (*GZMB*) to induce programmed cell death in the target cell (Trapani, 1995; Lopez et al., 2013). The immune checkpoint pathway includes the PD-1 axis (the receptor *PD-1*, and its ligands *PD-L1* and *PD-L2*), the CTLA-4 axis (the receptor *CTLA-4*, and its ligands *CD80* and *CD86*), and several other genes (e.g., *LAG-3*, *TIM-3*, *BTLA*). While currently available agents mostly target the PD-1 and CTLA-4 pathways, agents targeting other immune checkpoints such as LAG-3, TIM-3, BTLA are under active clinical development (Pardoll, 2012).

**FIGURE 1:**
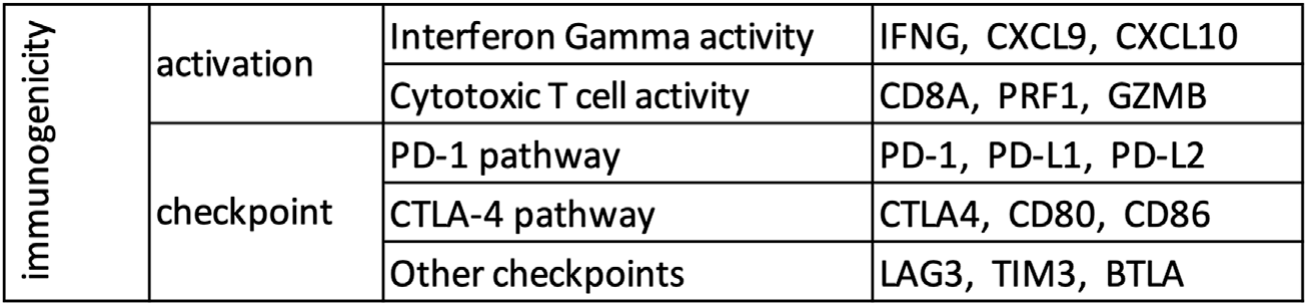
A small (15 genes) biologically motivated panel.

Therefore, in this study, we constructed a small biologically motivated panel, consisting of these 15 genes (Figure 1) involved in immune activation (6 genes) and checkpoint pathway (9 genes), and evaluated its usefulness for early identification of future responders to ICB.

## Methods

### Source of Data

RNA-seq data of tumor and adjacent normal samples from the Cancer Genome Atlas (TCGA) was obtained from NCI GDC (v4) Data Portal (https://portal.gdc.cancer.gov), and RNA-seq data of true normal samples from Genotype-Tissue Expression project (GTEx) was obtained from GTEx (v6p) Portal (https://www.gtexportal.org/).

### Activation Score, Checkpoint Score, and Immunogenicity Score

Both TCGA and GTEx datasets were restricted to 18,669 protein coding genes common in the two datasets using Ensembl BioMart (https://www.ensembl.org/biomart/martview/), and single sample gene set enrichment analysis (ssGSEA) (Barbie et al., 2009) as implemented in the ESTIMATE package (Yoshihara et al., 2013) was used to calculate the enrichment levels of various gene sets. Activation score, checkpoint score, and immunogenicity score were defined as the enrichment level of the 6 immune activation genes (Figure 1), 9 immune checkpoint genes (Figure 1), and all 15 genes respectively.

### Quantification of Immune Infiltration and CD8^+^ T Cell Infiltration

For each tumor in the TCGA dataset, the mRNA expression data was divided by the median value per sample, and then used to quantify immune infiltration and leukocyte composition by ESTIMATE (Yoshihara et al., 2013) and CIBERSORT (Newman et al., 2019) respectively.

### Cancer Subtypes Analyzed Separately

Subsequently, the mRNA expression data of tumors from the TCGA dataset was log transformed as x -> log_2_(1 + 1023*x), and breast cancer samples were classified into clinical subtypes based on the mRNA expression of *ESR1* and *ERBB2* (Supplementary Figure S1). The 3 clinical subtypes of breast cancer were analyzed separately, as were esophageal adenocarcinoma and esophageal squamous cell carcinoma, and pheochromocytoma and paraganglioma.

### Quantification of Pathology-Based Lymphocyte Infiltration

A pathologist scored >300 tumors from 10 cancer types in TCGA for the presence of tumor infiltrating lymphocytes in a blinded manner as previously described (Panda et al., 2017).

### Validation Dataset

Normalized nanostring data of metastatic melanoma patients undergoing ICB (i.e., “on-treatment”, not “pre-treatment”) was collected from the supplementary material (Tables S6a and S9c) of a recent study (Chen et al., 2016). Expression data of *BTLA* was not available for these patients, and only the data from Table S6a was used if a patient was present in both tables.

### Control Analysis

15 genes were randomly chosen from the non-immune genes common among the analyzed datasets and used as control (*AMIGO1*, *CDH1*, *CYCS*, *EFNA3*, *EFNA5*, *EPHA7*, *NFKBIE*, *NKIRAS1*, *P4HA2*, *S100A5*, *S1PR2*, *SDHA*, *SEMA3F*, *SEMA4B*, *SEMA5A*). Various analysis performed for the genes in our panel (Figure 1) were repeated for this control gene set.

### Statistics

Wilcoxon rank-sum test was used for all pairwise comparisons, and Pearson coefficient was used for all correlation analysis. All *p*-values are from two-sided tests, and *p* < 0.05 was used as the threshold for statistical significance.

## Results

### Consistency Check

**FIGURE 2:**
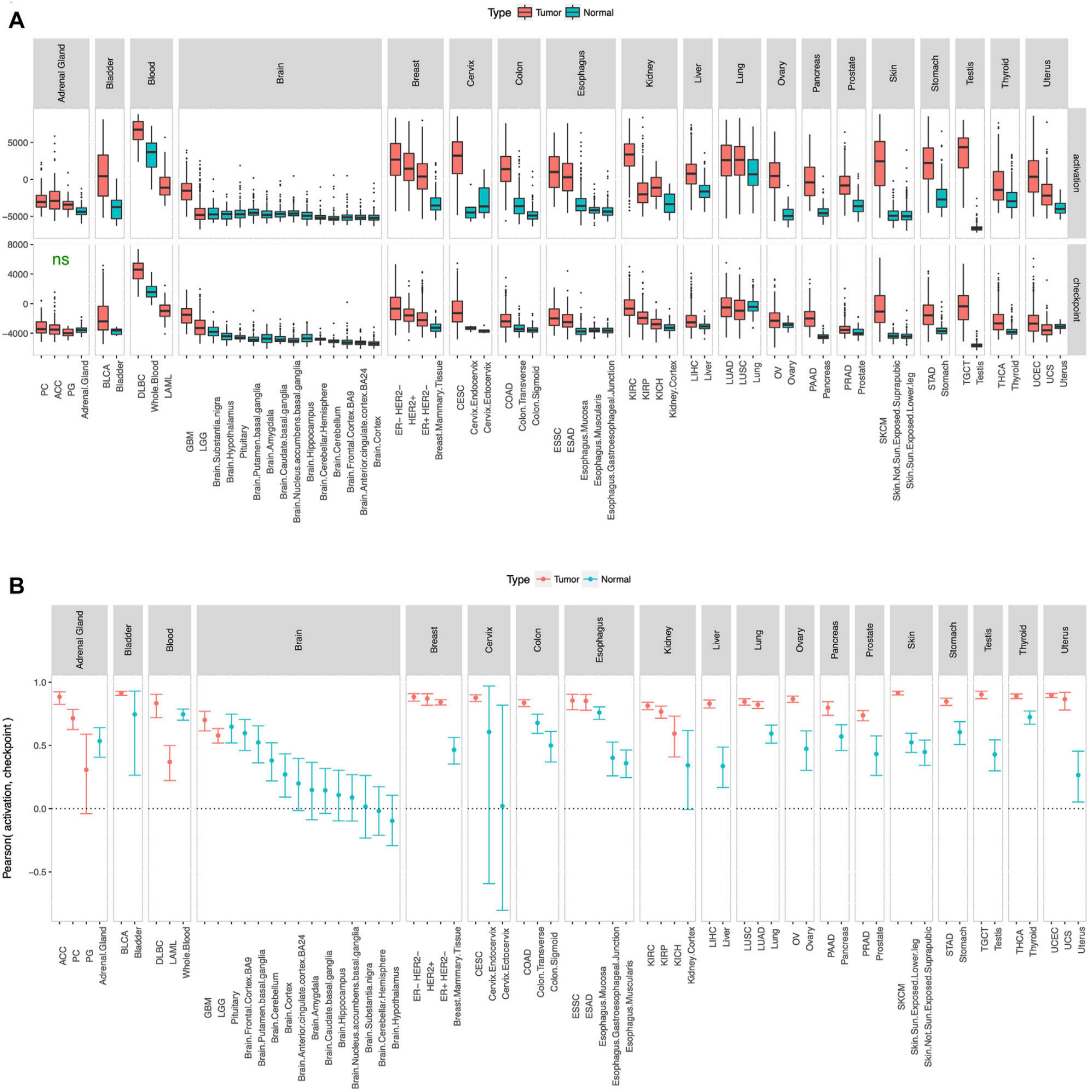
Consistency check. Activation score and checkpoint score **(A)** were substantially higher and **(B)** showed substantially stronger correlation in tumor than normal in almost every tissue type. Cancer type acronyms are standard TCGA abbreviations (https://gdc.cancer.gov/resources-tcga-users/tcga-code-tables/tcga-study-abbreviations) except esophageal adenocarcinoma (ESAD) and esophageal squamous cell carcinoma (ESSC), pheochromocytoma (PC) and paraganglioma (PG), and the 3 subtypes of breast cancer. ns = difference between tumor and normal not statistically significant.

To check whether the gene list in our panel (Figure 1) is reasonable, we defined activation score and checkpoint score as the ssGSEA (Barbie et al., 2009) enrichment level of the 6 immune activation genes and the 9 immune checkpoint genes respectively, and calculated these scores in tumor and adjacent normal samples from TCGA and true normal samples from GTEx (GTEx Consortium, 2013). Tumor adjacent normal samples from TCGA had substantially higher activation score and checkpoint score than true normal samples from GTEx in most tissue type (Supplementary Figure S2), which suggests that tumor adjacent normal samples are not truely normal at least from an immunological point of view. Therefore, for consistency check of the gene list in our panel (Figure 1), we compared tumor samples from TCGA and true normal samples from GTEx (Figure 2). As expected, in almost every tissue type, tumor samples had substantially higher activation score and checkpoint score than true normal samples (Figure 2A). Since tumor cells are more “non-self” than normal cells, tumor samples attract more immune infiltration than normal samples, hence the usually higher activation score in tumor samples than normal samples. Tumor samples usually have higher checkpoint score than normal samples most probably because tumor cells preferentially use the checkpoint pathways to block the anti-tumor immune response, more so than normal cells which also use the checkpoint pathways to avoid autoimmunity. Activation score and checkpoint score were significantly correlated in almost every tissue type even in normal samples (Figure 2B), as even normal samples use checkpoint pathways to block immune response and avoid auto-immunity; but the correlation was substantially stronger in tumor than normal in almost every tissue type (Figure 2B), suggesting that tumors preferentially use the checkpoint pathway to block immune response. Tumor cells over-expressing PD-L1 and other ligands merely prevents CD8^+^ T-cells from killing the tumor cells, potentially inducing T-cell exhaustion, but does not eliminate T-cell infiltration, and they remain stationed near the tumor cells. Also, in some cases PD-L1 is expressed by both tumor cells and infiltrating immune cells. Hence a high checkpoint score does not lower the activation score, instead a high checkpoint score and a high activation score usually occur together. Activation score and checkpoint score were very strongly correlated (Pearson coefficient >0.5) in every cancer type except paraganglioma and acute myeloid leukemia (Supplementary Figure S3), making it unnecessary to have two separate scores in the remaining cancer types. Therefore, we excluded these two cancer types from further analysis, and defined a single immunogenicity score as the ssGSEA (Barbie et al., 2009) enrichment level of all 15 genes in the remaining cancer types. The immunogenicity score was almost perfectly correlated (Pearson coefficient >0.99) with the sum of activation score and checkpoint score (Supplementary Figure S4), and well-correlated with the expression of each of the 15 genes in all cancer types (Supplementary Figure S5).

### Pathological and In-Silico Validation

**FIGURE 3:**
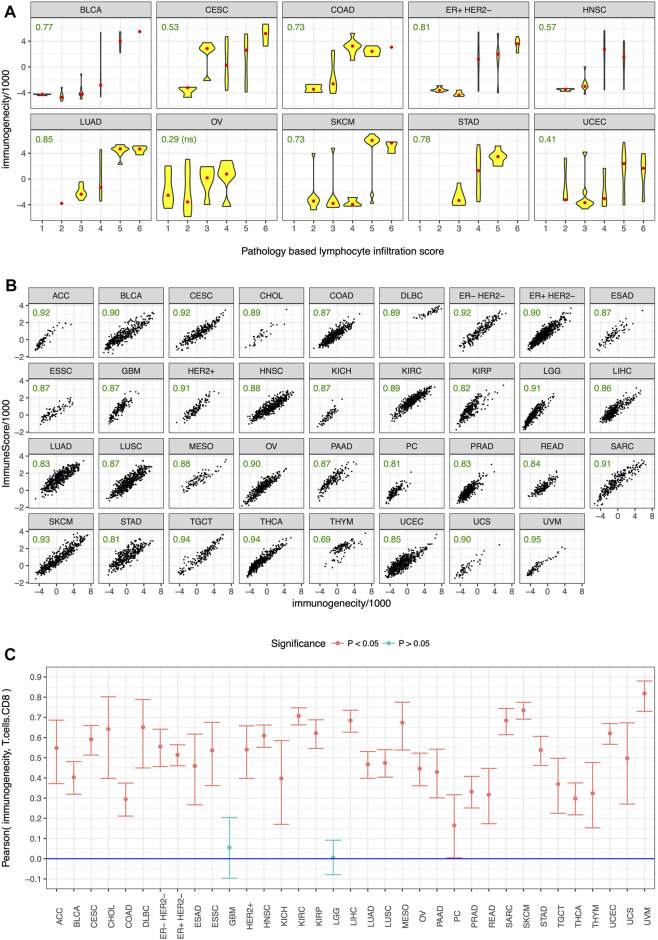
Pathological and in-silico validation. Immunogenicity score was correlated with **(A)** pathology-based lymphocyte infiltration score in almost all of the tested cancer types, and in-silico estimates of **(B)** overall immune infiltration and **(C)** CD8^+^ T cell infiltration in almost all cancer types. Cancer type acronyms are standard TCGA abbreviations (https://gdc.cancer.gov/resources-tcga-users/tcga-code-tables/tcga-study-abbreviations) except esophageal adenocarcinoma (ESAD) and esophageal squamous cell carcinoma (ESSC), pheochromocytoma (PC), and the three subtypes of breast cancer. Pearson correlation coefficients are specified in green in **(A,B)**, ns = not statistically significant.

To test the utility of our gene panel (Figure 1), we performed pathological and in-silico validation of the immunogenicity score in the TCGA dataset. We randomly chose 15 genes from the non-immune genes common among the analyzed datasets and used them as control. A pathologist scored >300 tumors from 10 cancer types for the presence of tumor infiltrating lymphocytes in a blinded manner as previously described (Panda et al., 2017), and tumors with strong lymphocyte infiltration, as evidenced by a high pathology-based lymphocyte infiltration score, also had high immunogenicity score (Figure 3A). No such correlation was observed between pathology-based lymphocyte infiltration score and enrichment level of the control genes (Supplementary Figure S6). For in-silico validation, the immunogenicity score was compared to the estimated levels of overall immune infiltration (Yoshihara et al., 2013) and CD8^+^ T-cell infiltration (Newman et al., 2019) in tumors. The immunogenicity score was very strongly correlated with overall immune infiltration in every cancer type (Figure 3B) and was significantly correlated with CD8^+^ T-cell infiltration in every cancer type except glioblastoma and low-grade glioma (Figure 3C). In contrast, enrichment level of the control genes was not correlated with overall immune infiltration (Supplementary Figure S7) and CD8^+^ T-cell infiltration (Supplementary Figure S8) in most cancer types and was often uncorrelated or anti-correlated instead. These results confirm that our gene panel (Figure 1) is a robust marker of immune infiltration in tumor.

### Early Identification of Future Responders

**FIGURE 4:**
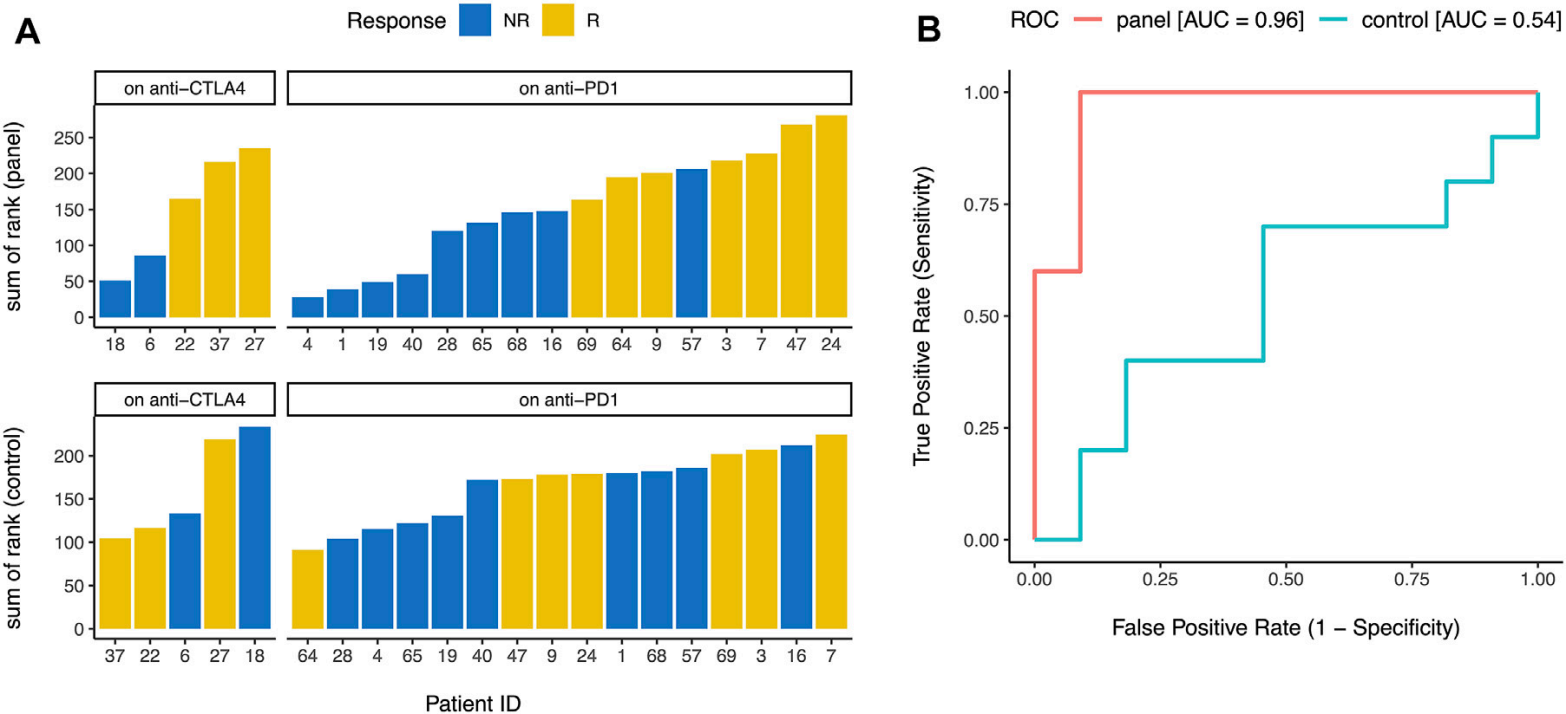
Early identification of future responders. **(A)** When metastatic melanoma patients on CTLA-4 blockade and PD-1 blockade were ranked by the normalized expression of each gene in the panel (Figure 1), the sum of these ranks accurately distinguished between future responders (R) and future non-responders (NR) (top). Similar analysis for 15 control genes did not distinguish between future responders and future non-responders (bottom). **(B)** Sum of ranks for the gene panel (Figure 1) was an excellent predictor of eventual response to immune checkpoint blockade (area under ROC curve = 0.96), but sum of ranks for 15 control genes was not predictive of eventual response to immune checkpoint blockade (area under ROC curve = 0.54). ROC = receiver operating characteristic, AUC = area under the curve.

Finally, we wanted to test whether our gene panel (Figure 1) can be used for early identification of future responders to ICB. Although ssGSEA (Barbie et al., 2009) enrichment levels (e.g., activation, checkpoint, and immunogenicity scores) are useful for consistency checks and in-silico or pathological validation of a gene list, calculating enrichment directly using this approach is usually not practical in individual clinical samples where whole transcriptome level data may not be easily available. So instead of calculating the enrichment level of the 15 genes in each sample, we ranked the samples by the normalized expression of each of the 15 genes (Figure 1) and then calculated the sum of these 15 ranks. In a publicly available dataset (Chen et al., 2016) of metastatic melanoma patients undergoing ICB (i.e., “on-treatment”, not “pre-treatment”), this sum of rank was an excellent predictor (area under ROC curve = 0.96 for our gene panel vs 0.54 for the control genes) of eventual response to CTLA-4 blockade and PD-1 blockade (Figure 4), confirming that our gene panel (Figure 1) can distinguish between future responders and future non-responders early in the course of ICB.

## Discussion

Immune checkpoint blockade (ICB) leads to durable responses in many cancer types (Kyi and Postow, 2016; Hargadon et al., 2018; Arora et al., 2019). Since a majority of patients do not respond to ICB (Yarchoan et al., 2017), it may be useful to identify the future responders early in the course of treatment. Previous work by our group (Mehnert et al., 2016; Panda et al., 2017; Panda et al., 2018a; Panda et al., 2018b; Panda et al., 2020) and others have identified several mechanism-based predictors of response to ICB, such as high tumor mutational burden (Mehnert et al., 2016; Panda et al., 2017), infection with exogenous viruses (Kwong et al., 2017; Panda et al., 2018b; Carbone et al., 2018), and expression of endogenous retroviruses (Panda et al., 2018a; Smith et al., 2018), and many additional mechanism-based predictors will likely be discovered in the future. Nevertheless, it is still useful to have a mechanism-agnostic tool for early identification of future responders, including those responders for whom the mechanism of response is yet to be discovered. Therefore, in this study, we evaluated a small (15 genes) biologically motivated panel (Figure 1) for early identification of future responders to ICB, consisting of 6 immune activation genes and the 9 immune checkpoint genes.

Our results show that ssGSEA (Barbie et al., 2009) enrichment level of the 6 immune activation genes and the 9 immune checkpoint genes were substantially higher (Figure 2A) and showed substantially stronger correlation (Figure 2B) in tumor samples from TCGA than normal samples from GTEx in almost every tissue type. ssGSEA (Barbie et al., 2009) enrichment level of the 15 genes was correlated with pathology-based lymphocyte infiltration score (Figure 3A) in almost all of the tested cancer types, and in-silico estimates of overall immune infiltration (Figure 3B) and CD8^+^ T cell infiltration (Figure 3C) in almost all cancer types. When metastatic melanoma patients on CTLA-4 blockade and PD-1 blockade were ranked by the normalized expression of each of these genes, the sum of these ranks was an excellent predictor (area under ROC curve >0.95) of eventual response to CTLA-4 blockade and PD-1 blockade respectively (Figure 4). Thus, our small biologically motivated panel (Figure 1) passed consistency check, pathological and in-silico validation, and was an excellent predictor of eventual response to CTLA-4 and PD-1 blockade when applied to patients on CTLA-4 and PD-1 blockade respectively.

While these results suggest that our gene panel (Figure 1) is useful for early identification of future responders to ICB, sum of ranks is not ideal for a clinical assay as ranking only helps when we have a group of samples. Developing the optimal method of using our gene panel to categorize individual samples without reference to a group of samples would be the next step towards a ready-to-use clinical assay. In addition to comparing the immunogenicity of different tumors and using it to select or prioritize patients for ICB, such an assay may also be useful for assessing or monitoring the immunogenicity of a tumor at various time-points during ICB, or for assessing the efficacy of experimental therapies designed to sensitize tumors to ICB. It may also be useful as a surrogate and/or substitute for pathological or in-silico estimate of immune infiltration in tumor. If calibrated using a sufficiently large dataset of patients treated with ICB, it may also be useful for quantifying the probability of response to ICB.

## Data Availability

The original contributions presented in the study are included in the article/Supplementary Material, further inquiries can be directed to the corresponding author.

## Supplementary Material

Supplementary Figure S1 Breast cancer samples were classified into the 3 clinical subtypes based on the mRNA expression of *ESR1* and *ERBB2*.

Supplementary Figure S2 Tumor adjacent normal samples from TCGA had substantially higher activation score and checkpoint score than true normal samples from GTEx in most tissue type. * *p* < 0.05

Supplementary Figure S3 Activation score and checkpoint score were very strongly correlated (Pearson coefficient >0.5) in every cancer type except paraganglioma (PG) and acute myeloid leukemia (LAML). Cancer type acronyms are standard TCGA abbreviations (https://gdc.cancer.gov/resources-tcga-users/tcga-code-tables/tcga-study-abbreviations) except esophageal adenocarcinoma (ESAD) and esophageal squamous cell carcinoma (ESSC), pheochromocytoma (PC) and paraganglioma (PG), and the 3 subtypes of breast cancer.

Supplementary Figure S4 Immunogenicity score was almost perfectly correlated (Pearson coefficient >0.99) with the sum of activation score and checkpoint score in all cancer types. Cancer type acronyms are standard TCGA abbreviations (https://gdc.cancer.gov/resources-tcga-users/tcga-code-tables/tcga-study-abbreviations) except esophageal adenocarcinoma (ESAD) and esophageal squamous cell carcinoma (ESSC), pheochromocytoma (PC), and the three subtypes of breast cancer.

Supplementary Figure S5 Immunogenicity score was well-correlated with the expression of each of the 15 genes in Figure 1 in all cancer types. Cancer type acronyms are standard TCGA abbreviations (https://gdc.cancer.gov/resources-tcga-users/tcga-code-tables/tcga-study-abbreviations) except esophageal adenocarcinoma (ESAD) and esophageal squamous cell carcinoma (ESSC), pheochromocytoma (PC), and the 3 subtypes of breast cancer.

Supplementary Figure S6 Control results for pathological validation. Enrichment level of 15 control genes was not correlated with pathology-based lymphocyte infiltration score in any of the tested cancer types. Cancer type acronyms are standard TCGA abbreviations (https://gdc.cancer.gov/resources-tcga-users/tcga-code-tables/tcga-study-abbreviations) except for ER+ HER2− breast cancer. Pearson correlation coefficients are specified in green, ns = not statistically significant.

Supplementary Figure S7 Control results for in-silico validation. Enrichment level of 15 control genes was not correlated with in-silico estimates of overall immune infiltration in most cancer types. Cancer type acronyms are standard TCGA abbreviations (https://gdc.cancer.gov/resources-tcga-users/tcga-code-tables/tcga-study-abbreviations) except esophageal adenocarcinoma (ESAD) and esophageal squamous cell carcinoma (ESSC), pheochromocytoma (PC), and the 3 subtypes of breast cancer. Pearson correlation coefficients are specified in green, ns = not statistically significant.

Supplementary Figure S8 Control results for in-silico validation (continued). Enrichment level of 15 control genes was not correlated with in-silico estimates of CD8^+^ T cell infiltration in most cancer types. Cancer type acronyms are standard TCGA abbreviations (https://gdc.cancer.gov/resources-tcga-users/tcga-code-tables/tcga-study-abbreviations) except esophageal adenocarcinoma (ESAD) and esophageal squamous cell carcinoma (ESSC), pheochromocytoma (PC), and the three subtypes of breast cancer.
