# Supplementary figures and images for: A Gene Panel for Early Identification of Future Responders to Immune Checkpoint Blockade
Source: Front Genet. 2022 Mar 3;13:706468. doi: 10.3389/fgene.2022.706468 (PMC8928072; doi:10.3389/fgene.2022.706468)

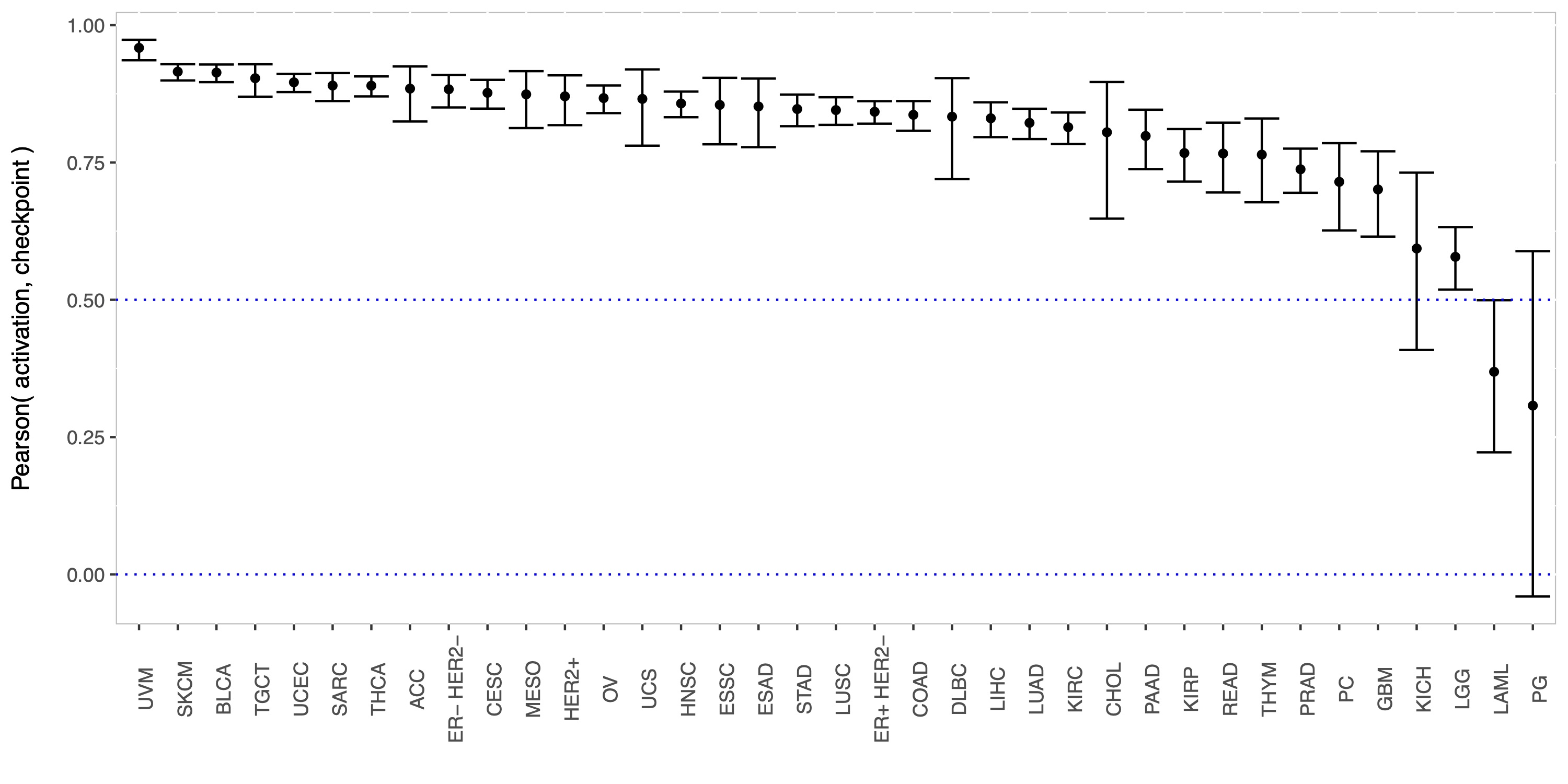

Supplement: Supplementary file 1 [file Image3.JPEG]

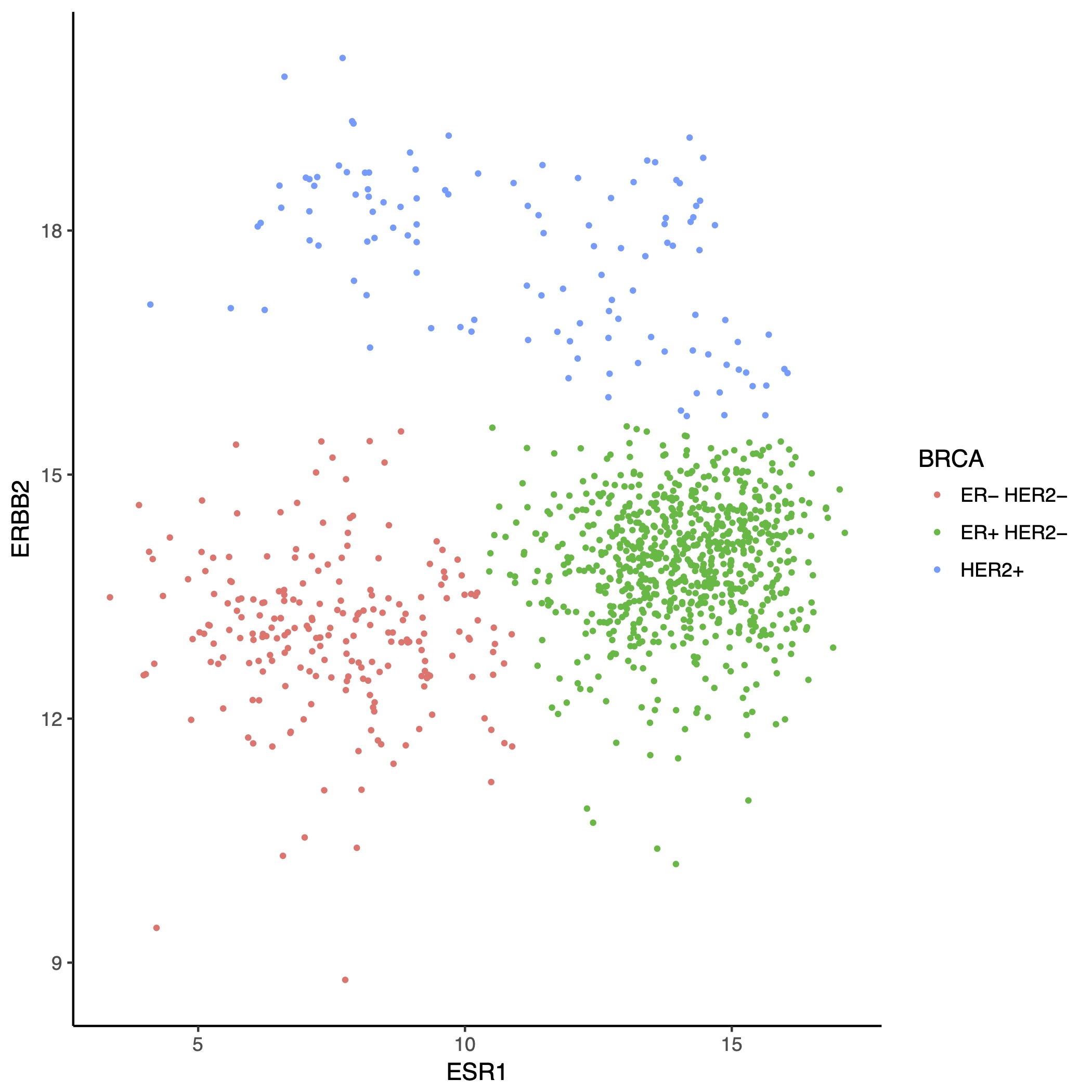

Supplement: Supplementary file 2 [file Image1.JPEG]

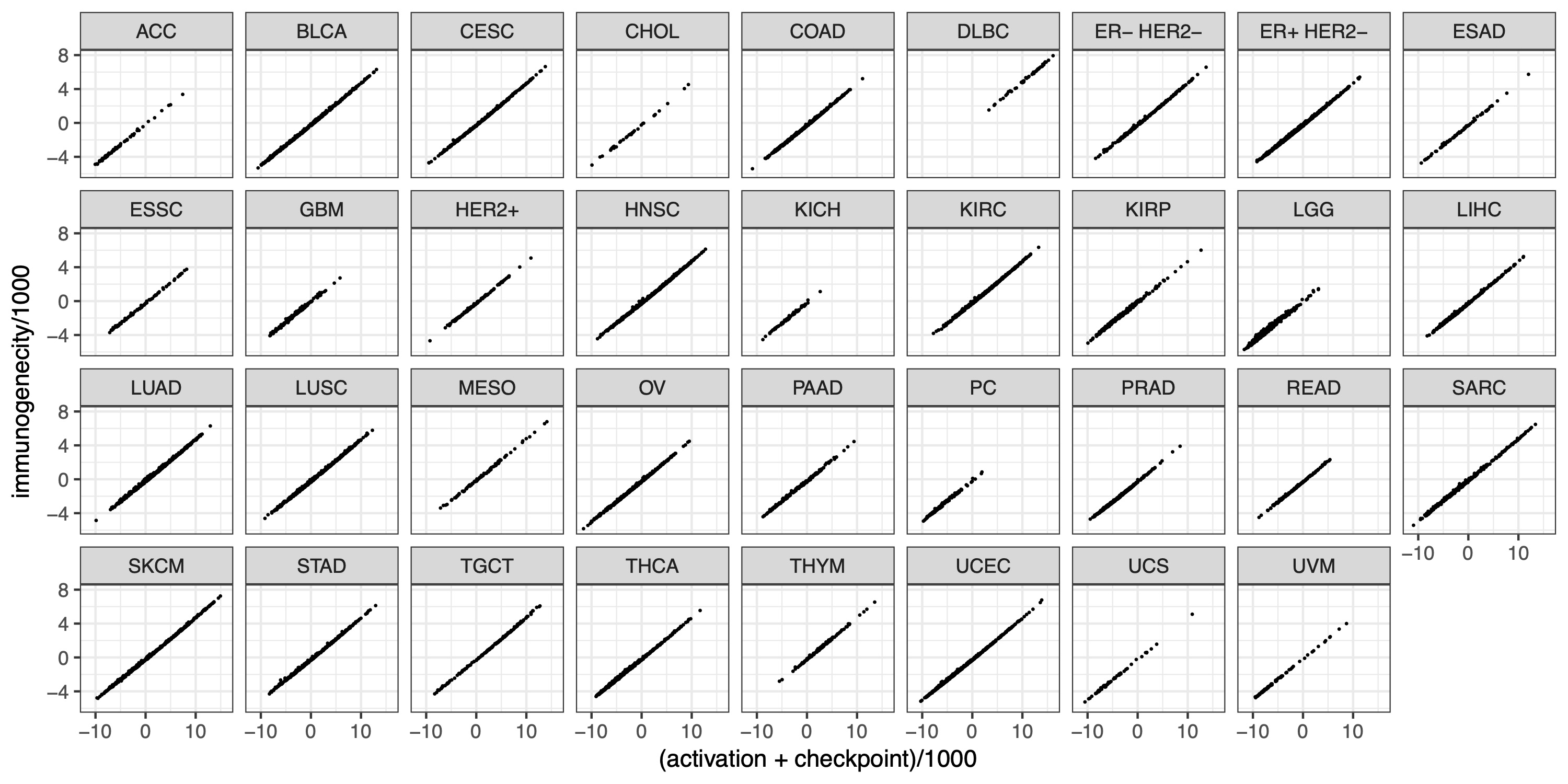

Supplement: Supplementary file 3 [file Image4.JPEG]

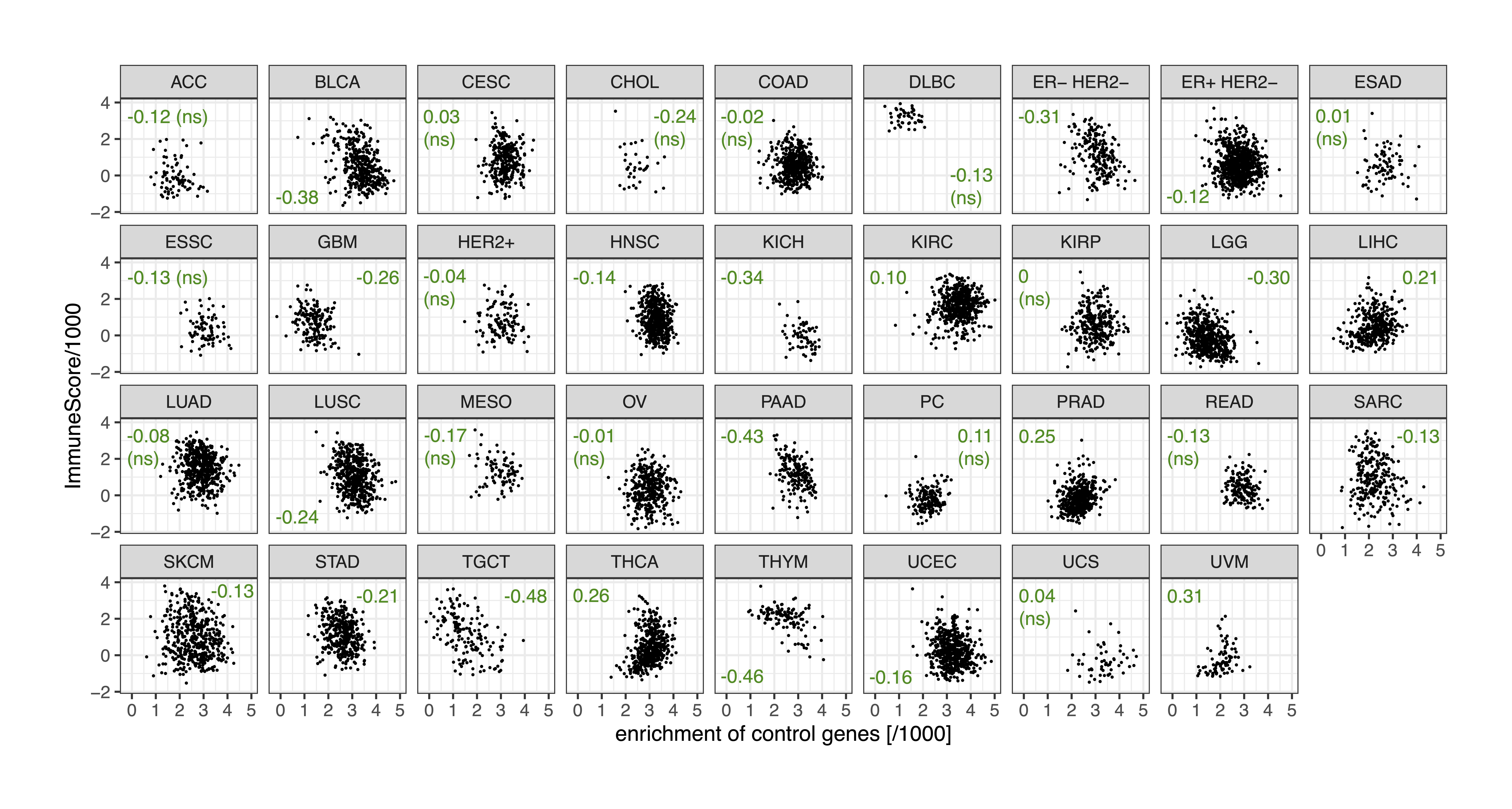

Supplement: Supplementary file 4 [file Image7.JPEG]

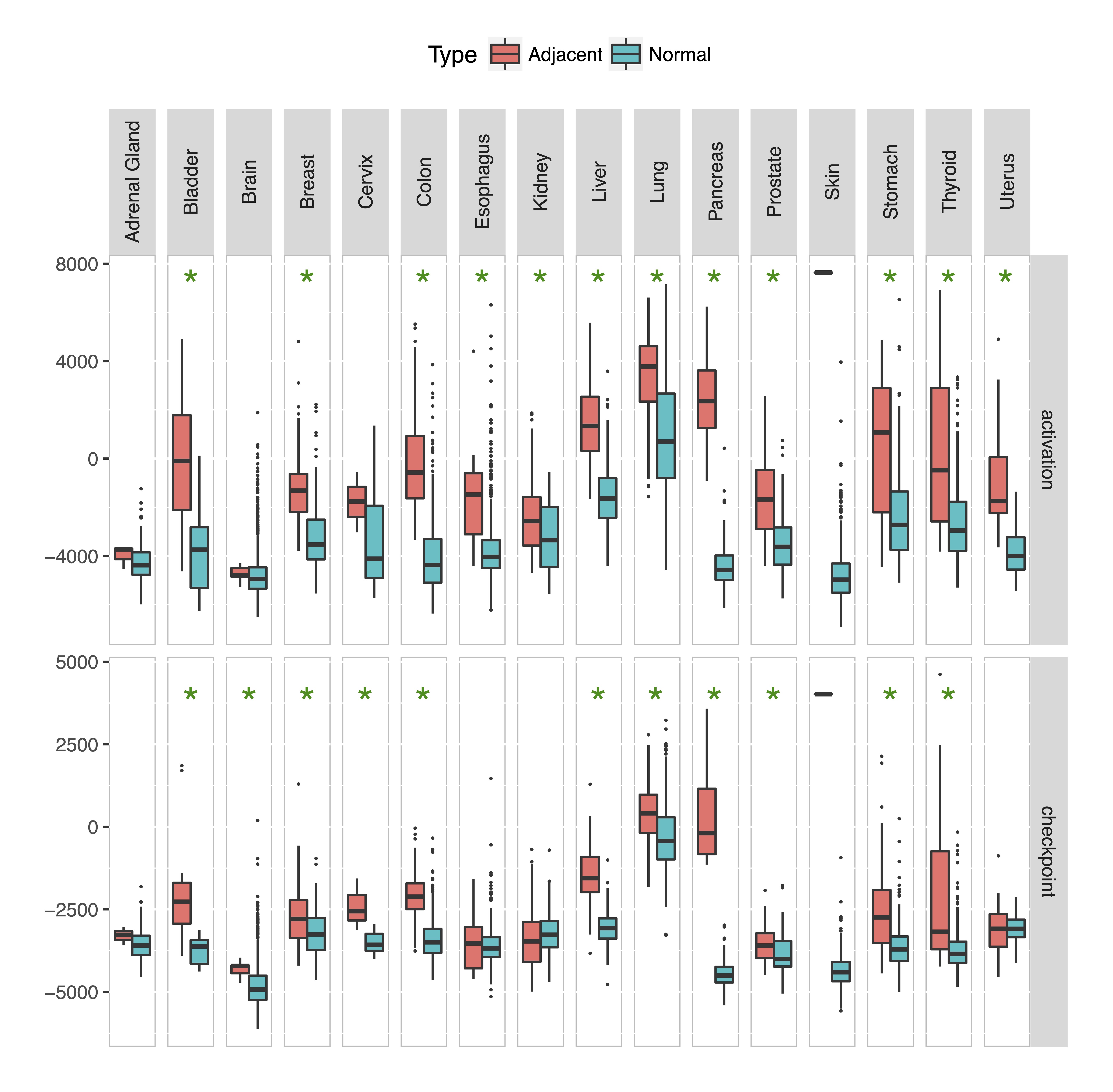

Supplement: Supplementary file 5 [file Image2.JPEG]

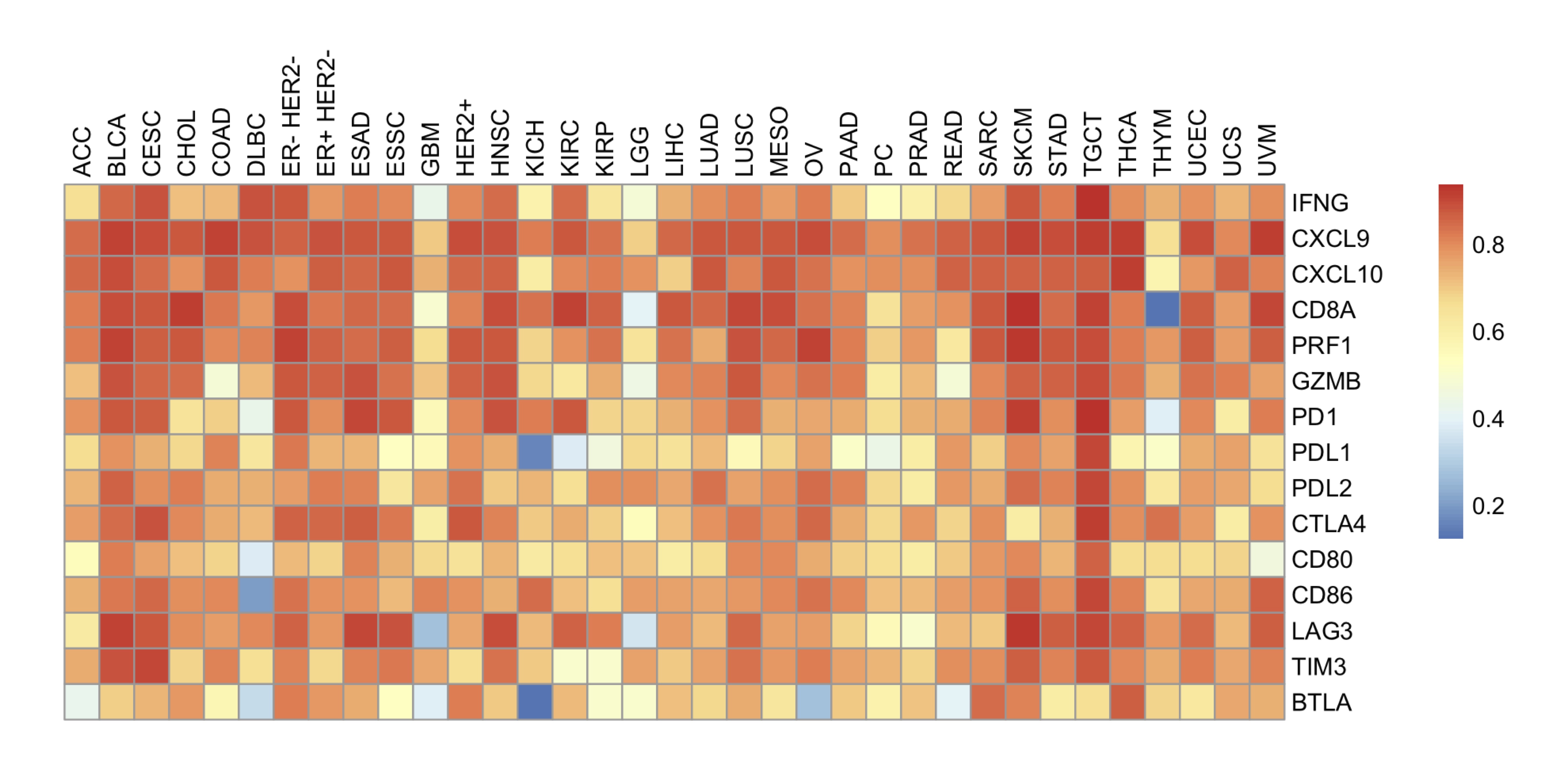

Supplement: Supplementary file 6 [file Image5.JPEG]

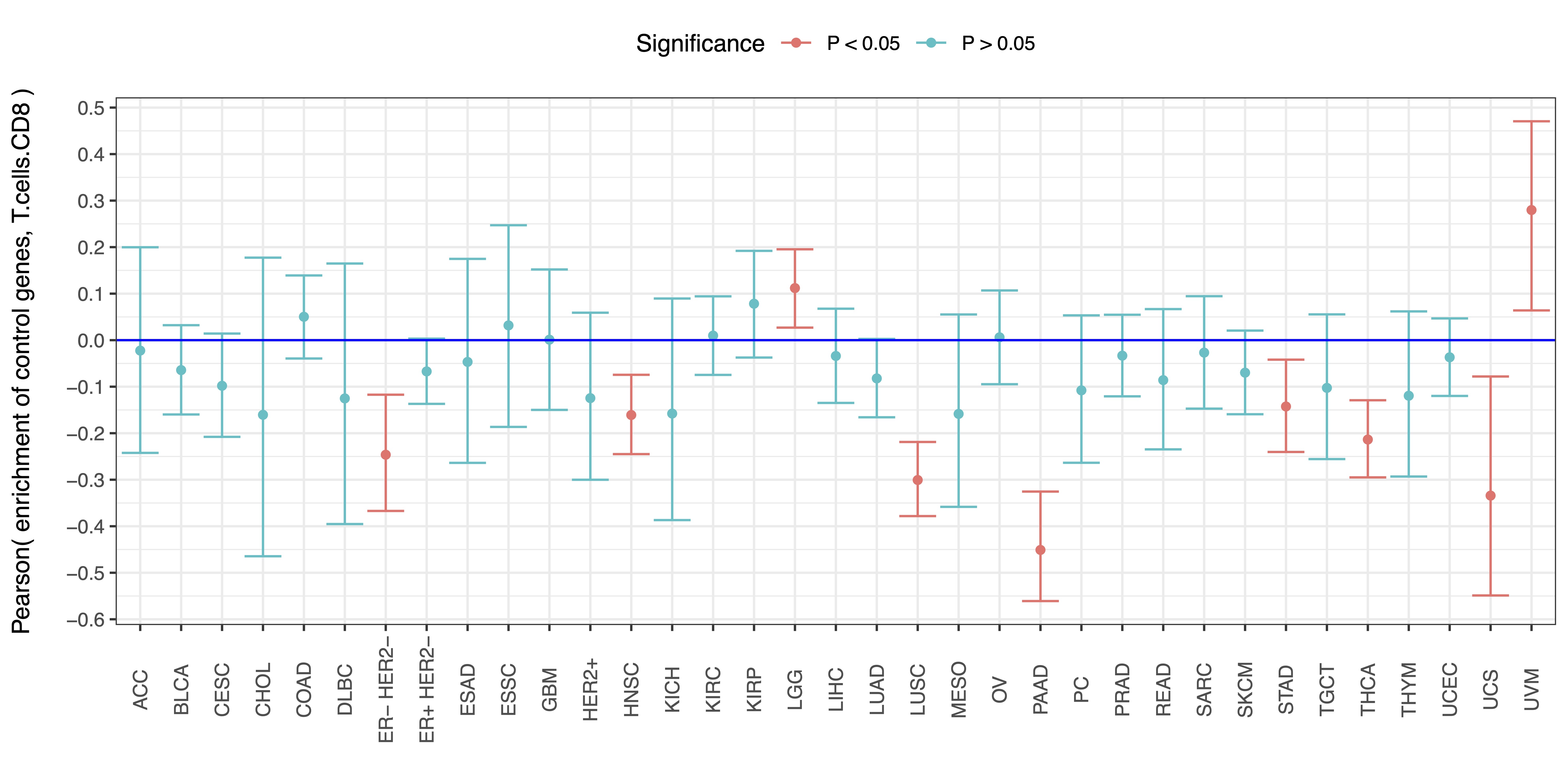

Supplement: Supplementary file 7 [file Image8.JPEG]

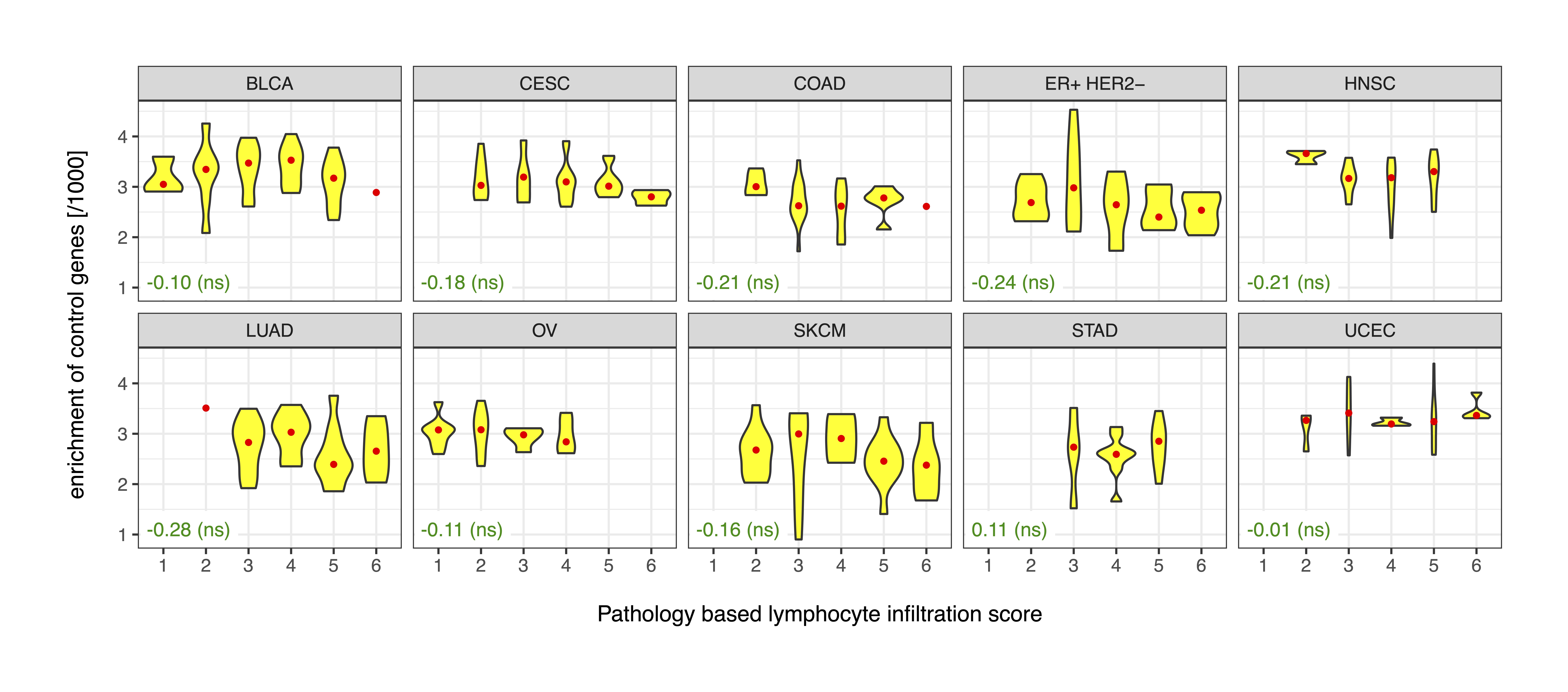

Supplement: Supplementary file 8 [file Image6.JPEG]
